# In vitro cytocompatibility of triclosan coated Polyglactin910 sutures

**DOI:** 10.1007/s10856-024-06796-w

**Published:** 2024-06-20

**Authors:** Hongrui Ji, Zhiruo Zhang, Chao Wang, Xuewen Li, Guiling Zhang, Danqing Liu

**Affiliations:** School of Materials Science and Chemical Engineering, Harbin University of Science and Technology, Harbin, 150040 Heilongjiang China

**Keywords:** Triclosan coated polyglactin910 sutures, Compatibility, Human umbilical vein endothelial cells, Hemolysis rate test, Cell proliferation test

## Abstract

Bioabsorbable sutures can improve the medical functions of existing non-absorbable sutures, and may produce new medical effects, and are expected to become a new generation of medical degradable materials. In this study, the cytocompatibility of triclosan coated polyglactin910 sutures (CTS-PLGA910) was analyzed and different concentrations of sutures were prepared. The effects of sutures on the cytotoxicity and cell proliferation of HUVEC were studied by CCK-8 assay. The hemolysis, total antioxidant capacity (T-AOC) activity and nitric oxide (NO) content were investigated to improve the blood compatibility of sutures. The results showed that the hemolysis rate of CTS-PLGA910 was less than 5%. After treatment on HUVEC cells for 48 and 72 h, there was no significant change in NO content in CTS-PLGA910 groups compared with the control group, while T-AOC activity and antioxidant capacity were significantly increased in medium and high dose groups. In summary, the blood compatibility and cell compatibility were significantly improved, which provided a basis for the clinical application of sutures in the future.

**Graphical Abstract:**

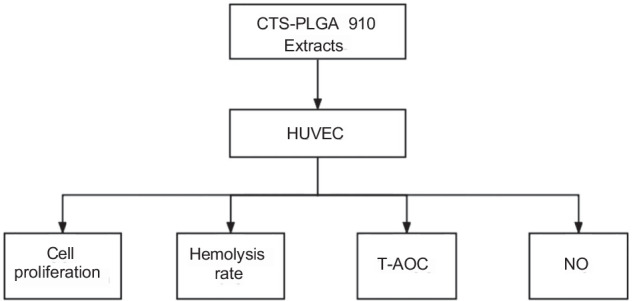

## Introduction

Bioabsorbable sutures are an important part of biodegradable materials [1]. It can shorten the healing time of sutured wounds, avoid the pain of taking out sutures again, and its application in medical clinic can greatly improve the medical function of existing non-absorbable sutures, and may produce new medical effects, thus bringing new Gospel to the majority of patients. It is of great significance in facial fine surgery such as stomatology, ent, and ophthalmology, and the drug loading of sutures plays an irreplaceable role in the treatment of wound sites [2].

PLGA [3] is a copolymer material obtained by the copolymerization of lactide (LA) and ethyl ester (GA) according to a certain ratio. It has good degradability and its degradation products are non-toxic. PLGA, as a coating material, is an important component of absorbable sutures and has a significant impact on the properties of sutures. It can not only improve the surface properties of sutures, but also affect the degradation rate.

Research and development of medical sutures with good biocompatibility is a topic of both social significance and economic value [4]. In this experiment, Polyglactin910 sutures coated with triclosan (CTS), CTS-PLGA910, were used. The compatibility of CTS-PLGA910 sutures with endothelial cells was investigated, and the total antioxidant capacity (T-AOC) and nitric oxide (NO) indexes were determined. It lays a solid foundation for its application in biomedical fields such as surgical sutures, fracture internal fixation materials, tissue repair materials and drug controlled release systems.

## Experimental materials and methods

### Experimental materials

CTS-PLGA910 and Human umbilical vein endothelial cells (HUVECs) were purchased from Aixikang Co., LTD. and Shanghai Meixuan Biological science and technology LTD respectively. Normal Blood was collected from the Adult Male Wistar rats (200 ± 20 g), which were purchased from Laboratory animal center (The Second Affifiliated Hospital of Harbin Medical University, China). The animals had free access to a commercial pellet diet and drinking water before all experiments.

All procedures were performed in this experiment had previously been approved by the School of Materials Science and Chemical Engineering, Harbin University of Science and Technology and all efforts were made to minimize suffering and the number of rats used.

### Hemolysis rate test

In the evaluation of biological materials, the evaluation of biocompatibility is the first. When the material is implanted in the body, it may react with red blood cells, causing them to rupture or release hemoglobin from the red blood cells through partial damage. The hemolysis rate test is one of the tests used to detect the blood compatibility of a material, and is used to determine the degree of erythrocyte lysis and hemoglobin release caused by the biological material in vitro.

In this experiment, 5 samples of CTS-PLGA910 were taken respectively, 10 mL normal saline was added into the test tube, and preheated at 37 °C for 30 min.The sample and 0.2 mL diluted blood were placed in a test tube and placed in a CO_2_ incubator at 37 °C for 60 min. Normal saline as negative control, three steamed water as positive control. All test tubes were centrifuged at 3000 rpm/min for 5 min. The supernate was taken and the absorbance of the supernate was measured at wavelength 545 nm with distilled water as reference.

The hemolysis rate was calculated according to the formula HR(%)= (DT-DNC)/(DPC-DNC) × 100%, where Dnc represents the absorbance of negative control, Dpc represents the absorbance of positive control, and Dt represents the absorbance of test samples.

According to the evaluation criteria of biological materials, when the hemolysis rate is less than 5%, it meets the requirements of medical materials for hemolysis rate. When the hemolysis rate is greater than 5%, it indicates that the biological material has hemolysis phenomenon, which cannot meet the requirements of medical materials, and the application of materials that directly or indirectly cause obvious hemolysis reaction will be limited.

### Preparation of the extract

In evaluating cytocompatibility, extracts can be used to examine. 1 g of treated sterile sutures were added into 20 mL of 1640 culture solution, placed in an incubator at 37 °C for 72 h, filtered with a microporous filter membrane (0.22 µl), and the prepared extracts were divided into high-concentration extracts, and then diluted with 1640 culture solution in equal proportion to prepare meddle-concentration extracts and low-concentration extracts respectively, and placed in a refrigerator at 4 °C for later use.

### Cell culture

HUVECs were cultured in RPMI1640 medium containing 10% fetal bovine serum, and 100 ug/mL streptomycin and 100 U/mL penicillin were added. Put in a CO_2_ incubator, 5% CO_2_, 37 °C static culture.

### Cell proliferation assay

CCK-8 can be used for cell proliferation and toxicity analysis. The basic principle is that the reagent contains WST-8, a compound similar to MTT that, in the presence of electron-coupled reagents, can be reduced by some dehydrogenase enzymes in the mitochondria to produce an orange-yellow methazan dye (formazan). The faster the cells proliferate, the darker the color; If the cytotoxicity is greater, the color is lighter. For the same cells, there is a linear relationship between the depth of the color and the number of cells.

In this experiment, CCK-8 kit was used to detect the cell proliferation and cytotoxicity of coated magnesium alloy. The specific steps of this experiment are as follows:

1. Cell suspension of 1 × 10^5^ cells/mL was prepared.
2. A cell suspension of 100 ul with the above cell concentration was inoculated into a 96-well plate 5 times for each sample, and normal cultured cells were used as a negative control. The 96-well plates were placed in a CO_2_ incubator and cultured for 24 h.
3. After cell adhesion culture, the original culture medium was replaced with different concentrations of extracts, and the culture was continued. At the detection time points of 24, 48 and 72 h, the CCK-8 kit of 10 µl was added to each hole. After about 4 h, the absorbance of each hole was measured at 450 nm by an immunoenzyme spectrometer, and the average value of the 5 repeated samples were calculated.

The relative cell proliferation rate was calculated by the following formula. Relative Growth Rate (RGR)=Average absorbance value of experimental group/average absorbance value of negative control group*100%. Results were then graded according to toxicity criteria specified in national safety standards [5] shown in Table 1.

**Table 1:** Grade evaluation of standard cell cytotoxity

Relative productivity of cells	≥100	75-99	50-74	25-49	1-24	<1
Toxicity grading	0	1	2	3	4	5

### Nitric oxide (NO) determination

NO is an extremely unstable biological free radical with small molecule, simple structure, little solubility in water, fat solubility, and rapid diffusion through biofilm. As a novel biological messenger molecule, nitric oxide plays a role in transmitting signals between cells and within cells. It also plays a very important role in the body’s nervous system, circulation, respiration, digestion, urogenital and other systems.

Different concentrations of extracts were used in this experiment. Firstly, a cell suspension with a concentration of 4 × 10^4^ cells /ml was prepared, and each well was 500 μl inoculated into a 24-well plate.

Normal cultured cells were used as the control group. After the cells were attached to the wall, the original culture medium was replaced with different concentrations of extracts. After 24 h of culture, the supernate of the cells was taken. The experimental procedure is according to the instructions of NO kit. The absorbance of each hole was determined at 570 nm. Absorbance was used to compare the NO content in each group of cells. The content of NO is proportional to the absorbance, and the greater the absorbance, the higher the content of NO.

### T-AOC determination

T-AOC refers to the total antioxidant level composed of various antioxidant substances and antioxidant enzymes. In order to protect cells and the body from oxidative stress damage caused by reactive oxygen species, total antioxidant capacity can be used to evaluate the antioxidant capacity of bioactive substances. Measuring the total antioxidant capacity of cell culture medium is of great significance for evaluating the antioxidant capacity of antioxidant substances.

A cell suspension with a concentration of 2 × 10^4^ cells /ml was prepared, and 1 ml of the cell suspension was inoculated into a 24-well plate with 5 replicates per well. Following the steps in the Total Antioxidant Capacity kit, the absorbance of each well is tested at 520 nm using an UV-visible spectrophotometer. The unit content of total antioxidant capacity was still positively correlated with absorbance, and the difference of total antioxidant capacity of each group was compared according to absorbance.

## Experimental results

### Hemolysis rate test results

CTS-PLGA910 extract did not show serious hemolysis phenomenon, and the hemolysis rate was 4.19%, lower than 5%, showing good blood compatibility and meeting the requirements of medical materials shown in Table 2.

**Table 2:** Results of hemolysis rate testing

Group	Absorbance	Hemolysis rate
CTS-PLGA910	0.1276 ± 0.0271	4.49%
Positive control group	0.7502 ± 0.0457	–
Negative control group	0.0983 ± 0.0215	–

### Cell proliferation and toxicity results

**Fig. 1:**
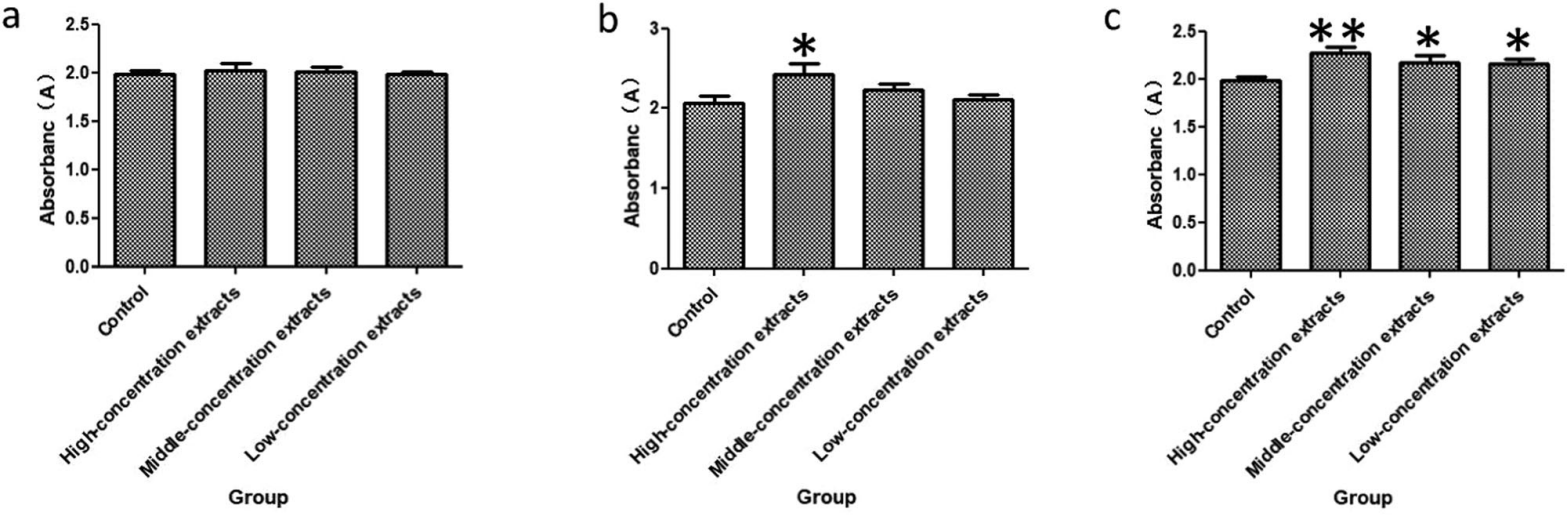
Proliferation of HUVECs cultured on extracts after 24h (**a**), 48h (**b**) and 72h (**c**) measured by CCK-8 Kits (Cell suspension of 1 × 10^5^ cells/mL was prepared. A cell suspension of 100 ul with the above cell concentration was inoculated into a 96-well plate 5 times for each sample, and normal cultured cells were used as a negative control. The 96-well plates were placed in a CO_2_ incubator and cultured for 24 h. After cell adhesion culture, the original culture medium was replaced with different concentrations of extracts, and the culture was continued. At the detection time points of 24 h, 48 h and 72 h, the CCK-8 kit of 10 μl was added to each hole. After about 4 h, the absorbance of each hole was measured at 450 nm by an immunoenzyme spectrometer, and the average value of the 5 repeated samples were calculated. The results of culturing for 24 h, 48 h and 72 h are showed as (**a**)–(**c**).**p < 0.01, *p < 0.05)

The effects of different concentrations of extracts on cell proliferation were compared by CCK-8 method. The proliferation results of cells cultured for 24, 48 and 72 h. Compared with the control group, the cells cultured for 24 h had no significant change in the three groups of high dose, medium dose and low dose shown in Fig. 1a, while the cells cultured for 48 and 72 h had significant increase in the high dose and medium dose extract group shown in Fig. 1b, c.

Cell culture for 24, 48, 72 h, the toxicity level is 0. In general, CTS-PLGA910 is safe. 48, 72 h after culture has a significant role in promoting the proliferation of HUVECS. The follow-up experiments were detected at high, middle and low dose groups of 48 and 72 h.

### Results of NO content

**Fig. 2:**
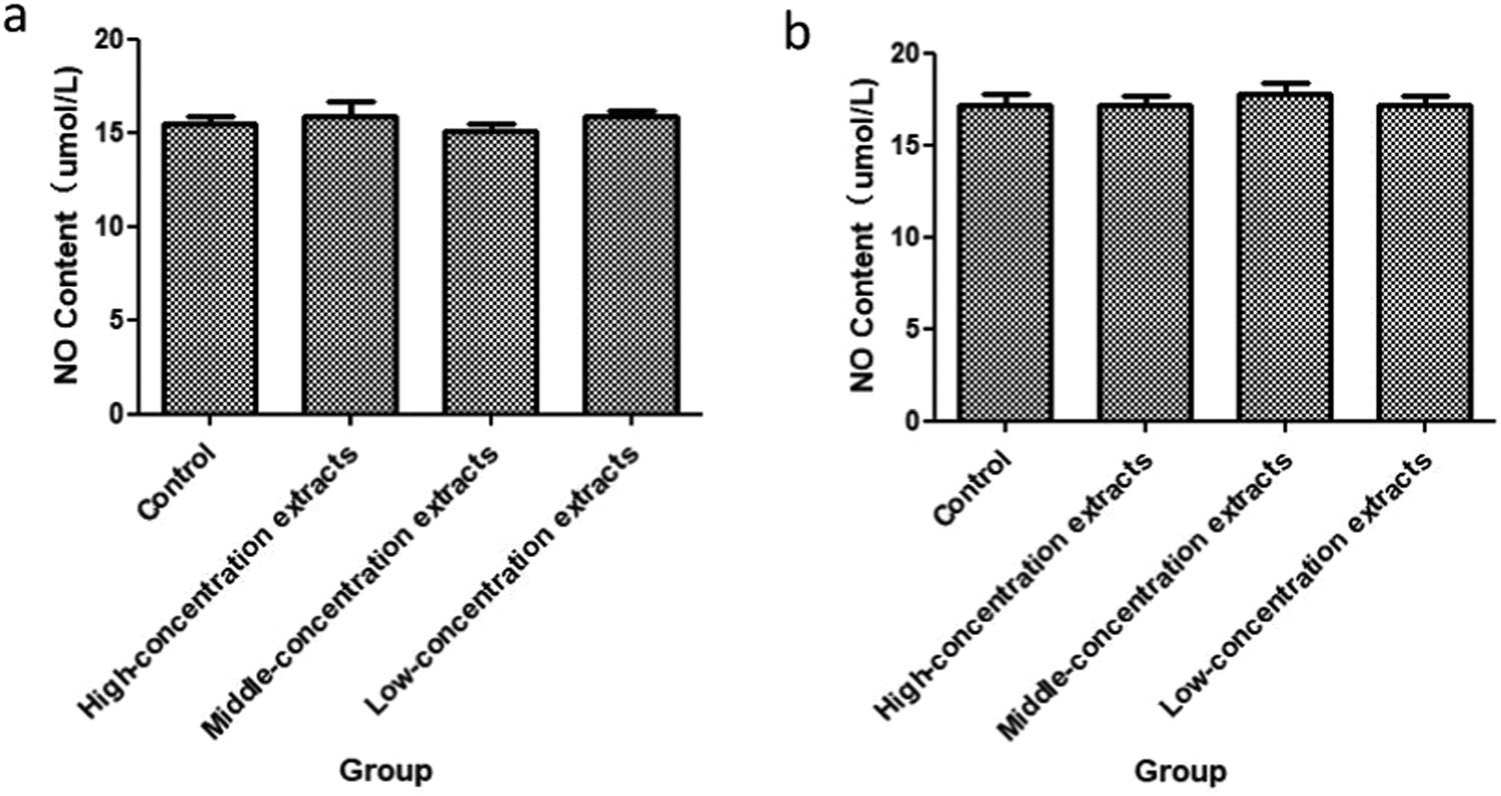
NO results of the different groups after HUVECs culturing for 48h (**a**) and 72 h (**b**) different concentrations of extracts were used in this experiment. Firstly, a cell suspension with a concentration of 4 × 10^4^ cells /ml was prepared, and each well was 500 μl inoculated into a 24-well plate. Normal cultured cells were used as the control group. After the cells were attached to the wall, the original culture medium was replaced with different concentrations of extracts. After 24 hours of culture, the supernate of the cells was taken. The experimental procedure is according to the instructions of NO kit. The absorbance of each hole was determined at 570 nm. Absorbance was used to compare the NO content in each group of cells. The content of NO is proportional to the absorbance, and the greater the absorbance, the higher the content of NO.The results of culturing for 48 h and 72 h are showed as (**a**), (**b**)

The absorbance value of NO was shown in Fig. 2. Compared with the control group, there was NO significant difference in the amount of NO released by cells in the high dose group and the medium dose group after culturing for 48 and 72 h, indicating that CTS-PLGA910 did not participate in the regulatory signal of angiogenesis [6].

### T-AOC determination results

**Fig. 3:**
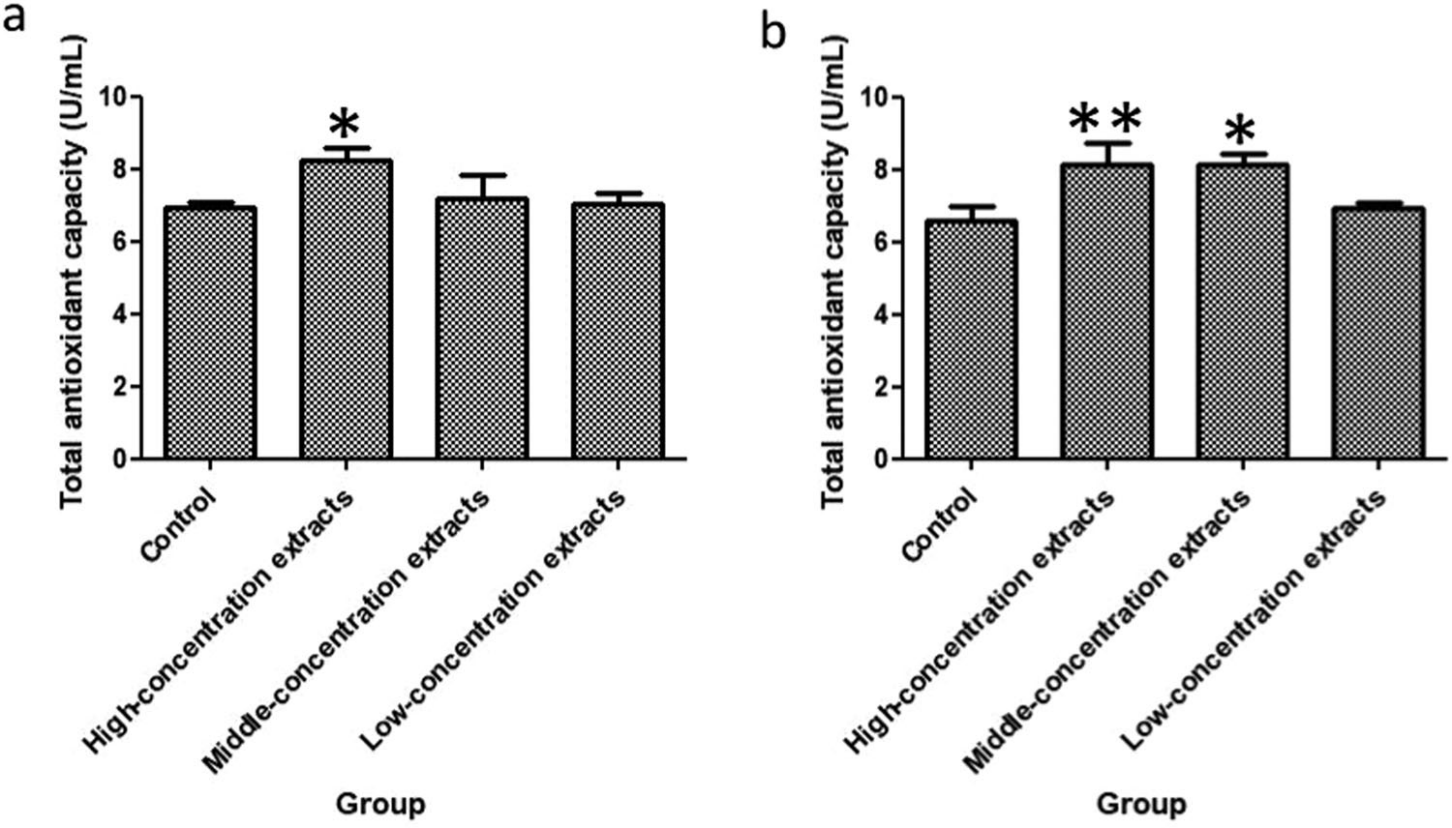
T-AOC contents of the different groups after HUVECs culturing for 48 h and 72 h (A cell suspension with a concentration of 2 × 10^4^ cells /ml was prepared, and 1 ml of the cell suspension was inoculated into a 24-well plate with 5 replicates per well. Following the steps in the Total Antioxidant Capacity kit, the absorbance of each well is tested at 520 nm using an UV-visible spectrophotometer. The unit content of total antioxidant capacity was still positively correlated with absorbance, and the difference of total antioxidant capacity of each group was compared according to absorbance. The results of culturing for 48 h and 72 h are showed as (**a**) and (**b**).**p < 0.01, *p < 0.05)

The normal physiological and metabolic processes of biological tissues and some environmental factors can induce the production of ROS. After the generation of reactive oxygen species, Oxidative damage of intracellular lipoproteins and DNA is caused, and Oxidative stress is induced, which then leads to various tumors, atherosclerosis, rheumatoid arthritis, diabetes, liver damage, and central nervous system diseases. Therefore, testing T-AOC of cells is often used to determine the antioxidant status of the body. The results of total antioxidant capacity are shown in Fig. 3.

## Conclusions

After sutures enter the human body, biocompatibility [7] is a relatively complex linkage process, and blood compatibility and cell compatibility are the two basic contents of evaluation. Especially for the suture material, its high corrosion rate may have a great impact on its biocompatibility [8].

The hemolysis rate of CTS-PLGA910 material is less than 5%, which meets the requirements of medical materials, and the material shows good blood compatibility.

Cytocompatibility [9] was studied by cell proliferation assay, cytotoxicity assay, NO assay and T-AOC assay. The effects of different concentrations of CTS-PLGA extracts on the proliferation of HUVECS were detected by CCK-8 kit. The results showed that different concentrations of extracts had different effects on endothelial cells. The effect of CTS-PLGA on the proliferation of endothelial cells did not change significantly after 24 h. After 48 and 72 h culture, the high concentration and medium concentration of the extract can promote the proliferation of endothelial cells. The cytotoxicity test showed that there was no obvious toxic reaction at 24, 48 and 72 h.

NO [10] is a functional gas produced by vascular endothelial cells and diffuses into vascular smooth muscle to relax it, thereby dilating blood vessels and lowering blood pressure. When it diffuses into platelet cells, its activity is reduced, and aggregation and adhesion are reduced to prevent the formation of thrombus. However, the amount of NO released has two sides, too much release will lead to the destruction of endothelial cell genes, resulting in cell damage or apoptosis. NO content was not significantly affected, and there was NO difference compared with the normal group, and the relative stability of NO level in the human body is favorable.

Culture 48, 72 h, high concentration, medium concentration extracts groups on the total antioxidant capacity of Huvecs have been greatly improved. In general, CTS-PLGA are widely used in the medical field due to their advantages of bioresorbability, adaptability, and safety. However, when choosing a medical suture, it is necessary to consider the patient’s specific situation and wound characteristics and make the choice according to the doctor’s recommendation.
